# Organization of the mitochondrial genome of *Ramulus irregulatiter dentatus* (Phasmatidae: Phasmatidae)

**DOI:** 10.3389/fgene.2022.967113

**Published:** 2022-08-29

**Authors:** Congfen Zhang, Xiaoqiang Guo

**Affiliations:** School of Pharmacy, Chengdu University, Chengdu, China

**Keywords:** phasmatodea, mitochondrial genome, *Ramulus irregulatiter dentatus*, gene organization, phylogenetic tree

## Abstract

Recently, the species of the insect order Phasmatodea, have attracted the interest of more and more enthusiasts. Here, we obtained the complete mitochondrial genome of *Ramulus irregulatiter dentatus* (*R. irregulatiter dentatus*), which belongs to the subfamily of Phasmatidae, detected by Illumina next-generation sequencing. The entire mitochondrial genome is 16,060 bp in length and contains a standard set of 13 protein-coding genes, 22 transfer RNA genes (tRNAs), 2 ribosomal RNA genes (rRNAs), and a putative A + T-rich region. The base composition and codon usage were typical of Phasmatodea species. The mitochondrial gene organization (37 genes) was consistent with that of other Phasmatidae. A phylogenetic tree was built from the sequence information of the 13 protein-coding genes by Bayesian analyses. The newly sequenced *R. irregulatiter dentatus* was most closely related to the family Phasmatidae. The complete mitochondrial genome of *R. irregulatiter dentatus* also provides valuable molecular information for future studies on Phasmatidae insect taxonomy and a framework to unveil more of their cryptic and unknown diversity, so that it can be used to control forest pests and protect crops.

## Introduction

The walking stick insects *Ramulus irregulatiter dentatus (R. irregulatiter dentatus),* which are noted for crypsis by phenotypically and behaviorally mimicking twigs, bark, lichen, moss, and leaves, are part of the subfamily Clitumninae (Phasmatidae: Phasmatidae) (Lee et al., 2018; Robertson et al., 2018). Most of them are characterized by long, dark brown or green colored bodies and feed on leaves of shrubs or trees, which provide them with the most efficient natural camouflage on Earth (Xu et al., 2021a). Stick insect species, often called walking sticks, are a mesodiverse group of large terrestrial insects inhabiting various habitats worldwide. Therefore, it is difficult to identify the phylogenetic relationships of Phasmatodea from the characterization of morphology. The walking stick insects, with more than 3,200 species in approximately 570 genera, are predominantly distributed in tropical regions and temperate areas, including Yunnan, Hunan, Guizhou and Sichuan Provinces in China.

Except for those few species of stick insects beneficial to humans (*E.g., Phyllium celebium, P. siccifolium* and *P. sinensis*), most of the walking stick insects are typical phytophagous pests, which are very harmful to their hosts (Xu et al., 2021b). An outbreak of these insects will erode a whole mountain forest and can even wreak havoc on surrounding crops. However, most studies of Phasmatodea have focused on taxonomy (Yang et al., 2021), biology (Gao et al., 2020), morphology (Everts, 2016), bionics (Pan et al., 2021; Kobayashi et al., 2022), prevention and control (Xu et al., 2021b; Yano et al., 2021), but the genera of Phasmatodea within the Polyneoptera family, and the evolutionary relationship between them remain unclear, particularly for highly diverse arthropods.

With the dramatic improvement of DNA sequencing innovation, the mitochondrial genome (mitogenome) groupings have been broadly utilized for recognizing the hereditary variety, species beginning and development, and molecular scientific categorization of insects because of their simple and stable gene composition, absence of genetic recombination, and effective evolution rate (Liu et al., 2016). Insect mitochondrial genomes ordinarily share similar elements with a 14–20 kb double-stranded circular molecule that consists of 37 genes (thirteen protein-coding genes, two ribosomal RNAs, and 22 transfer RNA genes) and a control region (CR). Mitochondrial genomes have been used for determining phylogenetic relationships among insect orders, such as Corydalidae (Zhang et al., 2020), Lepidopteran (Dai et al., 2015), Decapoda (Xin et al., 2018), and Phasmatodea (Xu et al., 2021a). Additionally, mitogenomes have been shown to be valuable in the investigation of phasmatodean molecular evolution, phylogenetics, phylogeography and population genetics (Plazzi et al., 2011; Bradler et al., 2014).

In this Phasmatidae research, we obtained the complete mitogenome sequence of *R. irregulatiter dentatus* and compared the structure and composition of the mitogenome with those of other available Phasmatidae mitogenomes. Additionally, to investigate the phylogenetic relationship from the perspective of mitogenomes and explain the phylogenetic location of *R. irregulatiter dentatus,* we constructed phylogenetic positions according to 13 PCGs of 20 Phasmatidae by Bayesian analysis theory and methods. This new investigation of the pool of Phasmatidae mitogenomes provides useful sequence information that can be helpful for distinguishing the species and understanding the evolutionary relationship of *R. irregulatiter dentatus* from a biological perspective, so that it can be used to control forest pests and protect crops.

## Materials and methods

### Sample collection

All samples used for sequencing in this study were collected from the Southern Sichuan Bamboo Sea in Sichun Province. The owner of the land gave approval to study on this site, and the work did not contain endangered or protected species. Insects were rinsed with saline, snap frozen in liquid nitrogen for capture and stored at −80°C until DNA extraction.

### DNA extraction, sequencing and gene annotation

Total genomic DNA was extracted from the whole body using the DNeasy Blood & Tissue Kit (Qiagen, Hilden, Germany). Subsequent high-throughput sequencing was performed by the Illumina NextSeq 500 Sequencing System (Illumina, CA, United States). The mitogenome was assembled using the SeqMan II program from the Lasergene software package (DNAStar Inc., Madison, United States). The beginning and stop codons of the protein-coding genes (PCGs) were determined by ORF Finder using invertebrate mitochondrial genetic codes, which was carried out by the NCBI website (https://www.ncbi.nlm.nih.gov/orffinder/). To acquire the base organization of nucleotide sequences, we determined composition skewness as depicted by Junqueira AT skew = [A−T]/[A + T], GC skew = [G−C]/[G + C]. The relative synonymous codon usage (RSCU) values were determined utilizing Codon W. The overlapping regions and intergenic spacers between genes were counted by manual means.

### Phylogenetic analysis

The nucleotide sequences of 20 insect whole mitochondrial genomes, or nearly complete mitochondrial genomes (nearly complete refers to complete coding genes), were obtained from NCBI, and the whole mitochondrial genome sequence was obtained by ClustalX version 2.0. The mitochondrial genomes were acquired from GenBank, and the GenBank accession numbers are presented in Table 1. The mitogenomes of *Drosophila incompta* (NC_025936) and *Trigoniophthalmus* alternatus (EU016193), Bombyx mori (MK295811), and *Atelura formicaria* (EU084035) were downloaded and used as outgroups. BI analyses were performed in MrBayes v3.2 [(Ronquist et al., 2012)] using the GTR+Γ substitution model. The runs were set for 50 million generations with sampling every 5,000 generations. Twenty-five percent of the aging samples were omitted, and the average standard deviation of split frequencies was below 0.01.

**TABLE 1:** Taxon sampling with accession numbers from NCBI.

Order/infraorder	Family	Species	Accession Number
Lonchodidae	Lonchodinae	*Megalophasma granula*tum	KY124331
		*Phraortes illepidus*	NC_014695
	Necrosciinae	*Micadina brachyptera*	MT025192
		*Micadina phluctainoides*	NC_014673
		*Calvisia medogensis*	KY124330
		*Neohirasea japonica*	AB477469
Phasmatidae	Platycraninae	*Megacrania alpheus adan*	NC_014688
	Eurycanthinae	*Dryococelus australis*	AP018522
		*Eurycantha calcarata*	NC_058255
	Clitumninae	*Pharnaciini sp. NS-2020*	MT025193
		*Phobaeticus serratipes*	AB477467
		*Ramulus hainanense*	FJ156750
		*Phryganistria guangxiensis*	MW450875
		*Entoria okinawaensis*	NC_014694
Bacilloidea	Bacillinae	*Bacillus rossius*	GU001956
	Heteropterygidae	*Orestes guangxiensis*	MW450873
		*Heteropteryx dilatata*	AB477468
Phyllioidea	Cryptophyllium	*Phyllium tibetense*	KX091862
Aschiphasmatoidea;	Aschiphasmatoidea;	*Nanhuaphasma hamicercum*	MZ312646
Pseudophasmatoidea	Pseudophasmatoidea	*Peruphasma schultei*	MW450874
		*Trigoniophthalmus alternatus*	EU016193
		*Drosophila incompta*	NC_025936
		*Bombyx mori*	MK295811
		*Atelura formicaria*	EU084035

## Results

### Mitochondrial genome organization and composition

The length of the complete mitochondrial genome of the walking stick insect *R. irregulatiter dentatus* was 16,060 bp. (Table 2), which is similar to a previous study (GenBank: AB477463.1). According to recent studies on the total mitochondrial genomes of stick and leaf insects, we found that the varying lengths of the Phasmatodea mitogenomes (15,590–18,248 bp) were caused by the size of the A + T-rich district, gene overlaps, and different intergenic nucleotides (IGNs). The mitogenome of *R. irregulatiter dentatus* (16,060 bp) contains a A + T-rich region of 1,465 bp. The mitochondrial genomes of the species had similar genes and gene organization as those of other reported stick insects, which have 37 genes, including 13 PCGs, 22 tRNA genes, and two rRNA genes. The gene organization pattern of the *R. irregulatiter* dentatus mitochondrial genome is similar to the assumed normal insect precursors (Xu et al., 2021c).

**TABLE 2:** Annotation and gene arrangement of the *Ramulus irregulatiter dentatus* mitogenome.

Gene	Direction	Location	Size (bp)	Anticodon	Start codon	Stop codon	Intergenic Nucleotides *
*trnI*	—	1–67	68	GAT		TA	−4
*trnQ*	+	65–133	79	TTG	TTA	TA	−2
*trnM*	—	133–199	67	CAT	AGA	—	5
*nad2*	—	203–1208	1006	—	ATC	TTA	−11
*trnW*	—	1209–1273	65	TCA		TA	−9
*trnC*	+	1266–1327	62	GCA		—	−1
*trnY*	+	1328–1390	83	GTA		TA	2
*cox1*	—	1392–2925	1534	—	ATG		−1
*trnL2(UUR)*	—	2926–2989	64	TAA		—	2
*cox2*	—	2990–3659	670	—	ATA	—	9
*trnK*	—	3660–3729	67	CTT		—	−2
*trnD*	—	3729–3795	76	GTC			−2
*atp8*	—	3796–3954	159	—	ATC	TAA	−5
*atp6*	—	3946–4625	678	—	ATG	—	8
*cox3*	—	4625–5413	789	—	ATG	TAA	4
*trnG*	—	5413–5477	65	TCC	ATT		2
*nad3*	—	5478–5829	352	—	ATT	T	6
*trnA*	—	5830–5894	65	TGC			−1
*trnR*	—	5895–5963	69	TCG			−1
*trnN*	—	5964–6027	64	GTT			−1
*trnS1(AGN)*	—	6027–6096	70	GCT			−1
*trnE*	—	6096–6159	64	TTC	ATT		0
*trnF*	+	6161–6223	63	GAA			3
*nad5*	+	6224–7946	1723	—	ATA	T	2
*trnH*	+	7947–8009	63	GTG		TT	−2
*nad4*	+	8009–9340	1332	—	TTA	T	−7
*nad4L*	+	9334–9624	291	—	TTA	T	2
*trnT*	—	9627–9688	62	TGT			−1
*trnP*	+	9689–9753	65	TGG		T	0
*nad6*	—	9755–10,234	480	—	ATA	TAA	5
*cob*	—	10,234–11,365	1132	—	ATG	T	2
*trnS2(UCN)*	—	11,366–11,433	68	TGA		TTA	−1
*nad1*	+	11,434–12,396	964	—	AAC	T	4
*trnL1(CUN)*	+	12,401–12,467	67	GTA	—	—	−31
*rrnL*	+	12,468–13,758	1258	—	—	T	2
*trnV*	+	13,759–13,826	68	TAC	—	—	0
*rrnS*	+	13,827–14,595	768	—	—	—	0
A+T-rich region		14,596–16060	1465	—	—	—	—

We found that the 13 PCGs of *R. irregulatiter dentatus were* 11,095 bp in length and accounted for 69.08% of the mitogenome length. Nine of these PCGs (*nad2, cox1, cox2, atp8, atp6, cox3, nad3, nad6,* and *cob*) were coded by the H-strand, while the other four PCGs (*nad5, nad4, nad4L,* and *nad1*) were coded by the L-strand. All PCGs started with the typical putative start codons (ATN), except for the *nad4* and *nad4L* genes with TTA. Five genes shared the complete termination codon TAA, while six genes adopted partial stop codons of a single T. A single T as a stop codon for *nad4* and *nad5* has been described in most of the sequenced Phasmatodea mitogenomes and even in some mammalian mitogenomes (Song et al., 2020). (Table 2).

**FIGURE 4:**
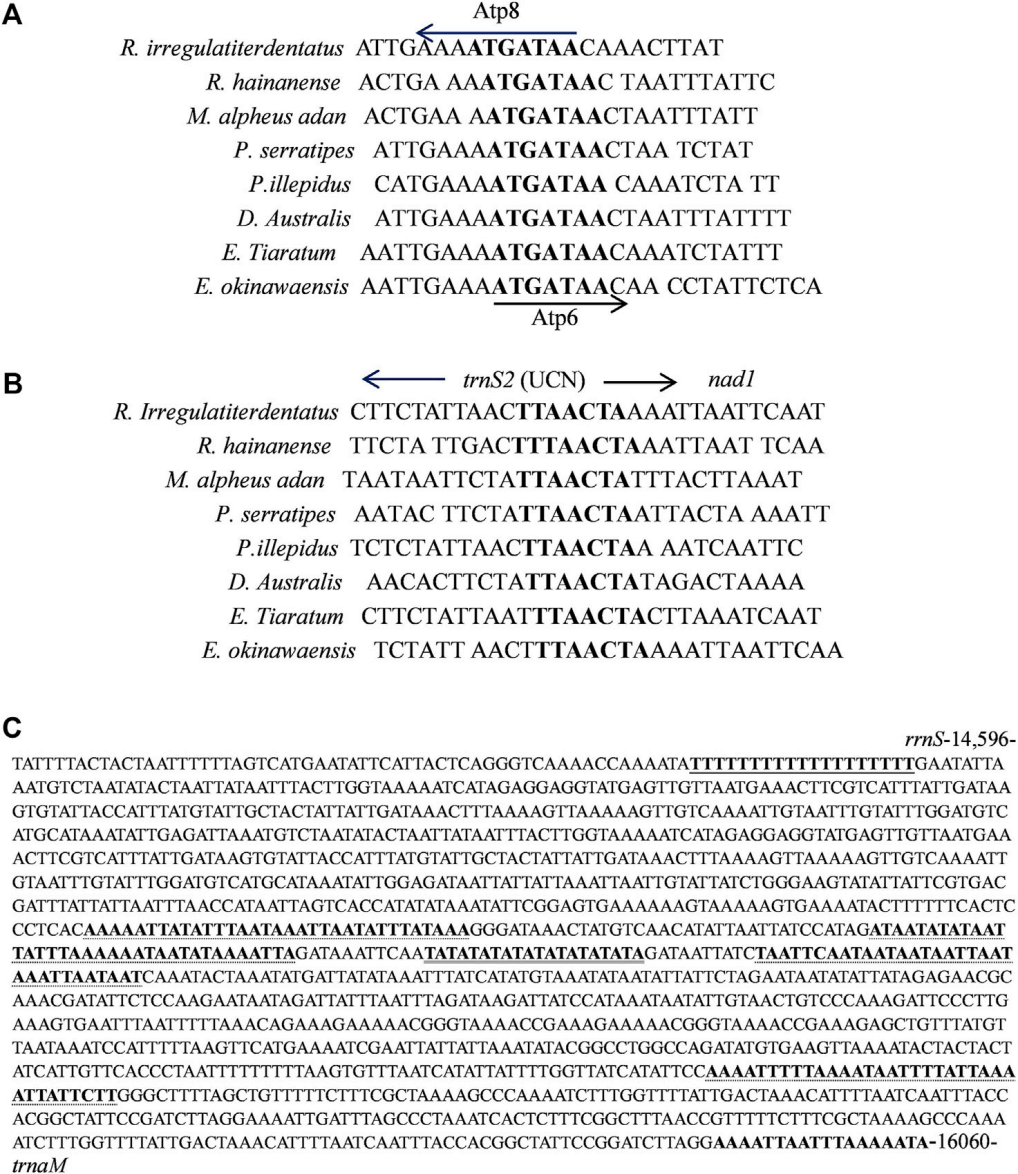
**(A)** Sequence analysis of the partial *Atp6* and *Atp8* regions of 8 Phasmatidae insects. The boxed nucleotides indicate the “ATAGATAA” conserved motif. **(B)** Sequence alignment of the partial *trnS2* and *nad1* regions of 8 Phasmatidae insects. The boxed nucleotides indicate the “TTACTA” conserved motif. **(C)** Features present in the A + T-rich region of *R. irregulariter dentatus*. The poly-T stretch is underlined, while the poly-A stretch is underlined in bold black letters. The single microsatellite T/A repeat sequence is indicated by double underlined, and long T/A repeats are indicated by dotted underlined.

Eleven overlapping sequences with lengths ranging from 1 to 31 bp were recognized in the *R. irregulatiter dentatus* mitogenome. The regions of 31 bp overlap were *trnL1*–*rrnL* 11 bp *nad2-trnW* and 9 bp *trnW*–*trnC* (Table 2). Other overlapping regions were shorter than 5 bp. The intergenic spacers of *R. irregulatiter dentatus* mitogenomes were distributed in 15 regions and varied in size from 1 to 9 bp with a total length of 58 bp. The longest spacer (9 bp) occurred between *cox2* and *trnK*. The 7 bp conserved sequence containing “ATGATAA” between *atp8* and *atp6* (Figure 4A) has also been documented in several Phasmatodea sequenced to date and the motif commonly exists in other insects, such as lepidopterans and megalopterans (Yang et al., 2020; Zhang et al., 2020). The intergenic spacers in *R. irregulatiter dentatus* were similar to those of the complete mitogenomes of other Phasmatodea insects (Song et al., 2020).

On the other hand, we identified the nucleotide composition of the *R. irregulatiter dentatus* mitochondrial genome. The frequencies of adenine, cytosine, guanine, and thymine were 37.5%, 9.34%, 15.07%, and 38.09%, respectively. A + T% shared 75% of the whole mitogenome length, which was similar to those of other previously reported phasmatodean mtDNAs (Kômoto et al., 2011). The A + T% values of 13 PCG, 22 tRNA and 2 rRNA genes accounted for 73.56%, 76.82%, and 81.66%, respectively. The 87.06% percent of A + T% in the A + T region was highest (Table 3). Thirteen PCGs and 2 rRNAs obtained negative scores, and 22 tRNA obtained positive scores. The estimated G + C skews and obtained negative scores were, similar to other species. All of the mtDNA sequenced in this study shared fairly common gene composition patterns among Hexapoda (Kômoto et al., 2011).

**TABLE 3:** Nucleotide composition of the *Ramulus irregulatiter dentatus* mitochondrial genome.

Feature	A%	C%	G%	T%	A+T%	A+T skew	G+C skew
Whole genome	37.50	9.34	15.07	38.09	75.60	−0.008	−0.235
PCG	30.81	12.68	13.77	42.74	73.56	−0.162	−0.041
rRNA	39.03	5.89	12.46	42.63	81.66	−0.044	−0.358
tRNA	38.96	9.55	13.63	37.85	76.82	0.014	−0.176
A+T region	38.81	3.98	8.96	48.26	87.06	−0.109	−0.385

### Protein-coding genes and codon usage

**FIGURE 1:**
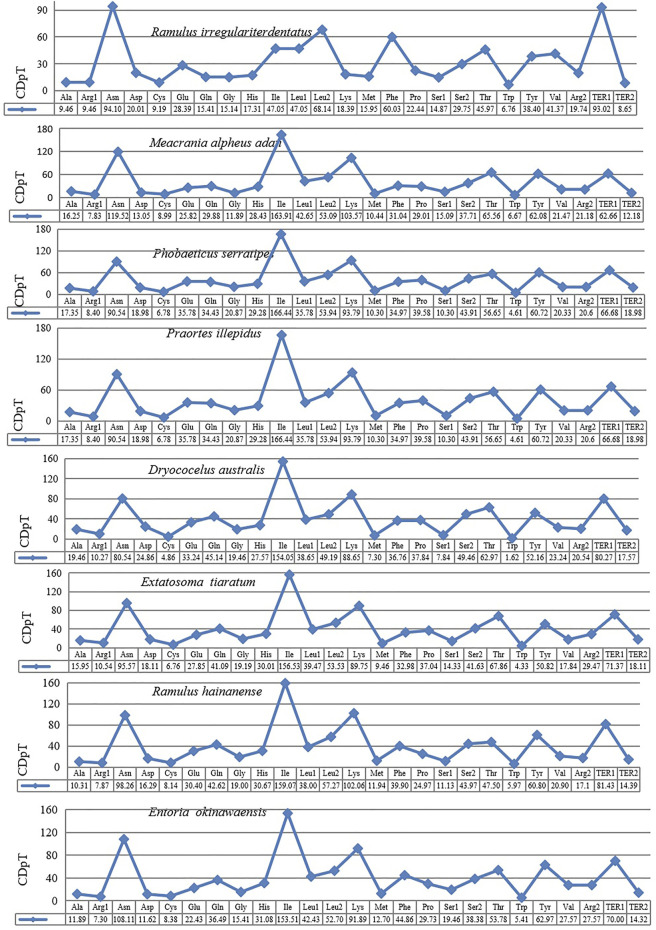
Codon distribution in Phasmatidae CDspT, codons per thousand codons. The protein-coding genes are from the following species: *Ramulus irregulariter dentatus*, *Megacrania alpheus adan* (NC_014688.1), *Phobaeticus serratipes* (NC_014678.1), *Praortes illepidus* (NC_014695.1), *Dryococelus australis* (AP018522), *Extatosoma tiaratum* (AB642680.1), *Ramulus hainanense* (FJ156750.1), and *Entoria okinawaensis* (NC_014694.1).

**FIGURE 2:**
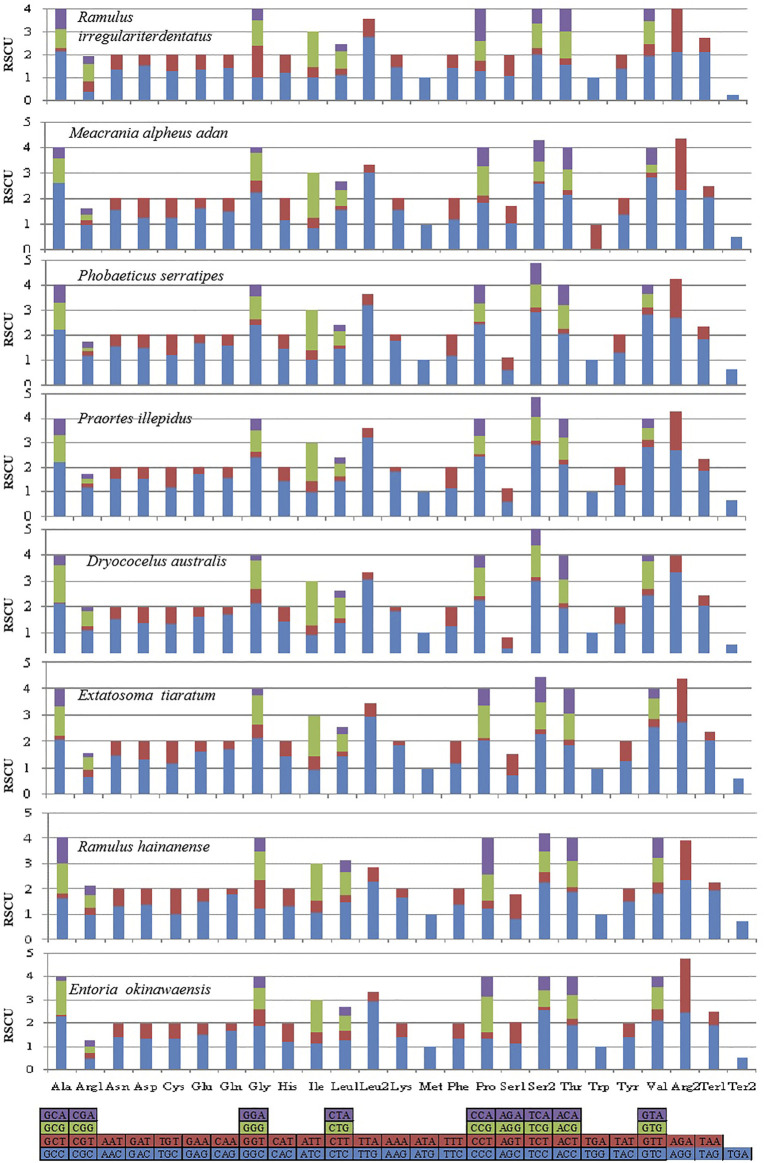
The mitochondrial genome (mitogenome) relative synonymous codon usage (RSCU) in Phasmatidae. Codon families are provided on the *X* axis. Codons indicated above the bar are not present in the mitogenome. The protein-coding genes are from the following species: *Ramulus irregulariter dentatus*, *Megacrania alpheus adan* (NC_014688.1*)*, *Phobaeticus serratipes* (NC_014678.1), *Praortes illepidu*s (NC_014695.1), *Dryococelus australis* (AP018522), *Extatosoma tiaratum* (AB642680.1), *Ramulus hainanense* (FJ156750.1), and *Entoria okinawaensis* (NC_014694.1).

The behavior of codon usage of the protein-coding genes in the *R. irregulatiter dentatus* mitochondrial genome in the PCGs was investigated. We found that all codons were present, and AUA (M), AAA (K), AAU (N), UUA (L), UAU (Y), AUU (I), and UUU (F) were the most abundant codons in the 13 PCGs (Table 4). Here, we compared the RSCU values among 8 PCGs calculated from the Phasmatidae mitogenomes (Figure 1). AGN codons (coding for Arg2), TAN (coding for Ter1), and TCN (coding for Ser2) were more frequently used than CGN codons (coding for Arg1), TGA (coding for Ter2), and AGN (coding for Ser1), respectively. Interestingly, TTN (coding for Leu2) was higher than CTN (coding for Leu1) in the mitogenomes of the 8 Phasmatidae insect species except *Ramulus hainanense.* From the codon distribution in Phasmatidae CDspT, we found that Asn and Ter1 were the richest. The CDspTs of Ile(47.0) and Lys(18.3) of the *R. irregulatiter dentatus* mitogenome are significantly lower than those of other Phasmatidae insects, but Leu2(68.1) and Phe(60) are the highest among them (Figure 2). This phenomenon is similar to that found in the mitogenomes of ditrysian (Wei et al., 2013), neuropterid (Enrico Negrisolo and Patarnello, 2011) and lepidopteran (Dai et al., 2016; Qiu-Ning Liu et al., 2016; Zou et al., 2017) insects.

**TABLE 4:** Codon usage of the protein-coding genes in the *Ramulus irregulatiter dentatus* mitochondrial genome.

Codon(aa)	No.	RSCU	Codon(aa)	No.	RSCU		Codon(aa)	No.	RSCU	Codon(aa)	No.	RSCU
UUU(F)	117	1.44	UCU(S)	30	1.10	UAU(Y)		193	1.37	UGU(C)	22	1.29
UUC(F)	45	0.56	UCC(S)	18	0.66	UAC(Y)		88	0.63	UGC(C)	12	0.71
UUA(L)	199	2.80	UCA(S)	55	2.01	UAA		263	2.10	UGA(W)	32	0.26
UUG(L)	53	0.75	UCG(S)	7	0.26	UAG		81	0.65	UGG(W)	25	1.00
CUU(L)	53	0.75	CCU(P)	18	0.87	CAU(H)		69	1.21	CGU(R)	14	0.78
CUC(L)	23	0.32	CCC(P)	29	1.40	CAC(H)		45	0.79	CGC(R)	6	0.33
CUA(L)	78	1.10	CCA(P)	27	1.30	CAA(Q)		110	1.41	CGA(R)	7	0.39
CUG(L)	20	0.28	CCG(P)	9	0.43	CAG(Q)		46	0.59	CGG(R)	8	0.44
AUU(I)	168	1.02	ACU(U)	49	1.15	AAU(N)		234	1.34	AGU(S)	29	1.06
AUC(I)	73	0.44	ACC(U)	43	1.01	AAC(N)		114	0.66	AGC(S)	25	0.91
AUA(M)	254	1.54	ACA(U)	67	1.58	AAA(K)		248	1.45	AGA(S)	38	2.11
AUG(M)	59	1.00	ACG(U)	11	0.26	AAG(K)		95	0.55	AGG(S)	35	1.94
GUU(V)	21	1.00	GCU(A)	7	0.80	GAU(D)		56	1.51	GGU(G)	16	1.14
GUC(V)	11	0.52	GCC(A)	8	0.91	GAC(D)		18	0.49	GGC(G)	7	0.50
GUA(V)	40	1.90	GCA(A)	19	2.17	GAA(E)		70	1.33	GGA(G)	14	1.00
GUG(V)	12	0.57	GCG	1	0.11	GAG(E)		35	0.67	GGG(G)	19	1.36

### Ribosomal RNA and transfer RNA genes

The mitochondrial genome of *R. irregulatiter dentatus* had 22 tRNA genes, ranging from 62 bp (trnC, trnT) to 83 bp (trnY), as in other Phasmatodea mitogenomes. Fourteen genes were encoded on the H-strand, and the others were encoded on the L-strand (Table 2). The total tRNA size of *R. irregulatiter dentatus* was 1,484 bp, with a high A + T bias of 76.8% (Table 3).

**FIGURE 3:**
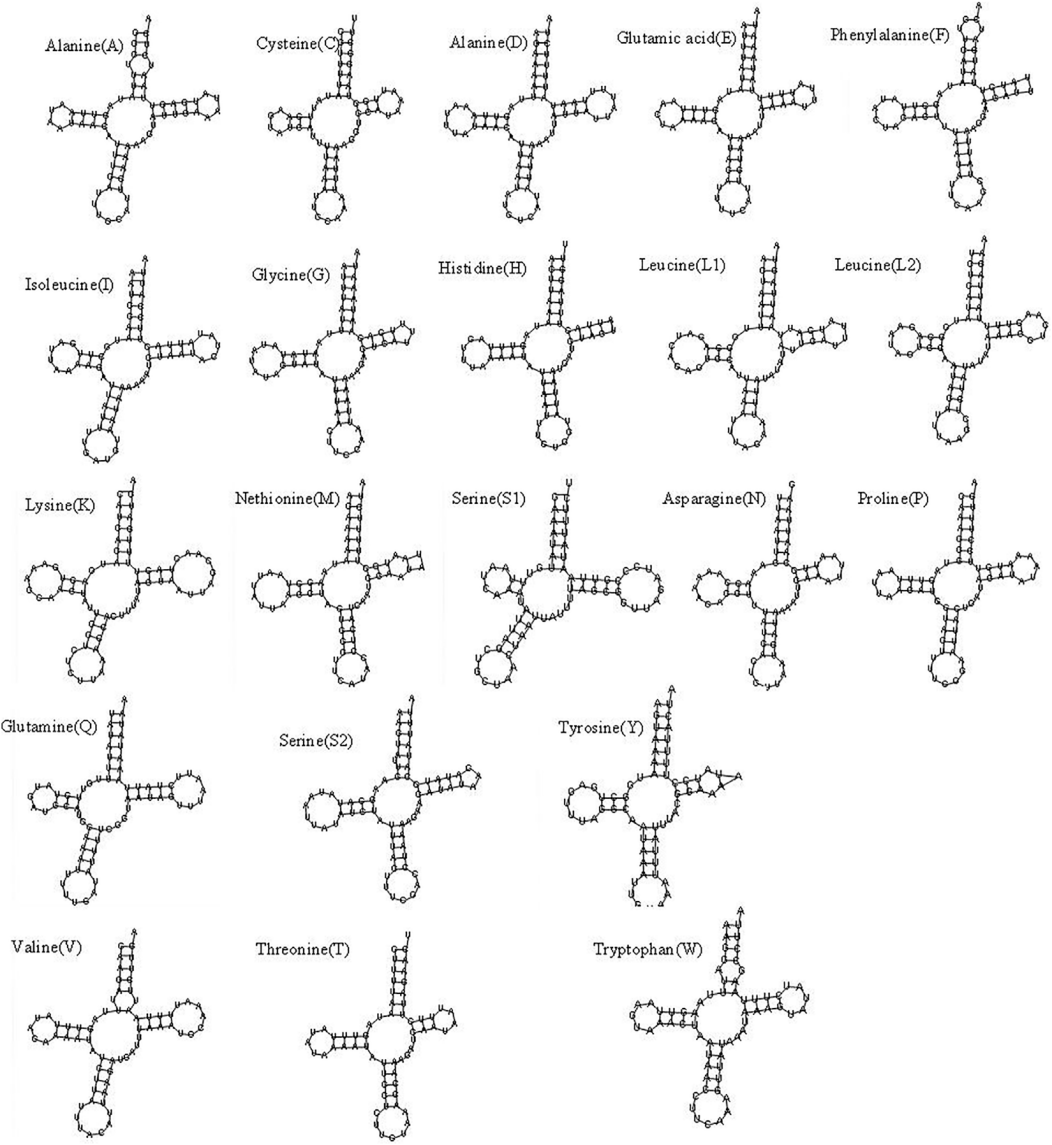
Putative secondary structures of the 22 tRNA genes of the *Ramulus irregulariter dentatus* mitogenome.

The AT skew was positive 0.014 compared with GC being negative 0.176 (Table 3). All tRNAs could be folded into normal secondary cloverleaf structures except for the *trnH*, *trnM* and *trnF* genes, which lack TΨC loops (Figure 3). This phenomenon was also observed in the mitochondrial genomes of the other three Phasmatodea insects, including *trnN* (*Orestes guangxiensis* and *Peruphasma schultei*) and *trnP* (*O. guangxiensis*) losing the TΨC loops, and *trnS1* (*O. guangxiensis*) lacking the dihydrouridine (DHC) arm (Xu et al., 2021a). Generally, in the absence of DHC arms or TΨC loops in other stick insects and other species of insects, there is lower translational activity compared with that of the typical structures (Wang et al., 2019; Zhang et al., 2020; Xu et al., 2021a). We also found some incorrect pairs, such as unmatched U-U base pairs in *trnV*, A-G base pairs in *trnW,* A-A base pairs in *trnS1*, A-G base pairs in *trnW*, C-A base pairs in *trnG*, and U-U base pairs in *trnV*, *trnA, trnY*, *trnS2*, and *trnL1,* in three of the other Phasmatodea insects (*O. guangxiensis*, *Peruphasma schultei*, and *Phryganistria guangxiensis*) (Xu et al., 2021a). There were 2 or 3 G-U pairs in *trn A*, *trn C*, *trn F*, *trn G*, *and trn H,* and one mismatched G-U base pair included *trnD, trnL2, trnK, trnM, trnS1, trnP, trnR, trnY, and trnT* (Figure 3). Most mismatched pairs were located at the amino acid acceptor stem and DHU stem of the transfer RNA gene secondary structures. Mismatched pairs may affect aminoacylation and the function of binding to ribosomes.

The 7 bp spacer sequence between *trnS2* and *nad1* contained the “TTAACTA” motif (Figure 4B), which is a common feature among Phasmatidae insects. Several similar motifs were found in other insects, including lepidopterans with “ATACTAA” (Yang et al., 2020) and pentanucleotide TCTAA conservative motifs existing in the overlap regions between COX1 and trnL2 of several other stick insects (Plazzi et al., 2011).

### The A + T-rich region

The mitogenome of *R. irregulatiter dentatus* includes an A + T-rich region of 1,465 bp that shares the highest A + T content (87.06%), negative AT skew (−0.109) and GC skew (−0.385) (Table 3). These A + T content values were similar to those found in the presented phasmatodean mitogenomes, such as 78.0% for *Pharnaciini spec. indet.*, 76.3% for *M. brachptera*, and 76.9% fo*r Phraortes* sp. (Song et al., 2020). The control region was characterized by a 19 bp poly-T stretch, a microsatellite-like (TA)_10_ and a terminal poly-A element (Figure 4C). Multiple tandem repeat elements, as a characteristic of the insect A + T-rich region, have been found in the mitogenome sequence of *R. irregulatiter dentatus.* The A + T-rich region having four sequences with more than 30 bp long repeats included “AAA​AAT​TAT​ATT​TAA​TAA​ATT​AAT​ATT​TAT​AAA,” “ATA​ATA​TAT​AAT​TAT​TTA​AAA​AAT​A ATATAAAATTA,” “TAA​TTC​AAT​AAT​AAT​AAT​TAA​TAA​ATT​AAT​AAT,” and “AAA​ATT​TTT​AAA​ATA​ATT​TTA​TTA​AAA​TTA​TTC​TT.” Copies of tandem repeats regions in the AT-rich region were also detected among other Phasmatodea mitogenomes including *Ramulus hainanense*, *Extatosoma tiaratum* and *Phraortes* sp. *1 NS-2020* (Shuichiro Tomita and Kômoto, 2011).

### Phylogenetic relationships

**FIGURE 5:**
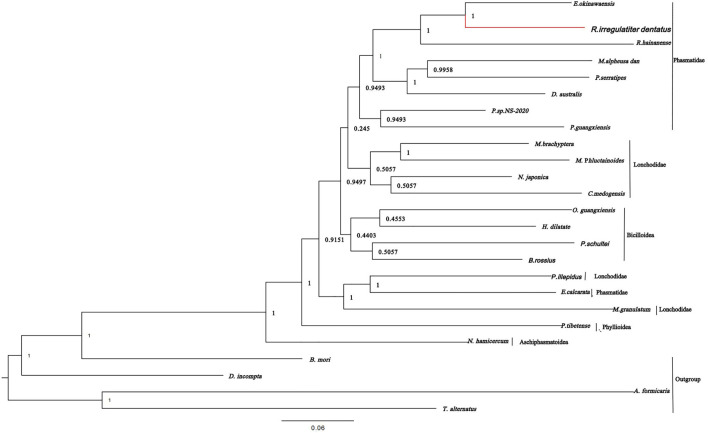
Phylogenetic relationships were built from the mitochondrial genome sequences of 20 insects by Bayesian inference methods.

According to the phylogenetic tree of *R. irregulatiter dentatus*, it can be confirmed that Lonchodinae, Necrosciinae, Platycraninae, Eurycanthinae, Clitumninae, Heteropterygidae, Aschiphasmatoidea, Pseudophasmatoidea, etc., are all on the same large branch. It revealed that *R. irregulatiter dentatus* is most closely related to *E. okinawaensis* and *R. hainanense* in the Clitumninae subfamily. In addition, Phasmatidae and Lonchodidae are the families that are most closely related, followed by Bacilloidea (Figure 5). In the present research, the relationships at the superfamily level are consistent with previous investigations of Phasmatodea family-level groups by wing venation characters. Our data suggested that mitochondrial genome sequences are evolutionarily conserved among different Phasmatodea species. Molecular phylogenetic analysis based on mitochondrial genomes can provide profoundly helpful information on the scientific classification of Phasmatodea.

## Discussion

The mitogenome of *R. irregulatiter dentatus* is a typical circular DNA molecule 16,060 bp long. The mitogenome sequence had the same genes and gene organization as those formerly investigated for other stick insect species, with 37 genes involving 13 protein-coding genes (PCGs), 22 tRNA genes, and two rRNA genes (Xu et al., 2021c). All protein-coding genes (PCGs) start with standard ATN initiation codons except for *nad4* and *nad4L*. Twenty-two tRNAs are predicted to fold into a characteristic secondary cloverleaf structure, except for the *trnH*, *trnM*, and *trnF* genes, which lack TΨC loops. The sizes of the large and small ribosomal RNA genes are 1,258 bp and 768 bp, respectively. The A + T control region contained a 19 bp poly-T stretch, a microsatellite-like (TA)_10_ and a terminal poly-A element. In addition, the phylogenetic analyses confirmed that *R. irregulatiter dentatus* belongs to Phasmatidae.

Phasmatodea, as a large group of mostly nocturnal herbivore insects, is regarded as a potential forest pest not only in China but also in Korea, Japan and North America (Lee et al., 2018; Liu, 2021; Yano et al., 2021). In China, stick insects have defoliated trees and damaged more than 60 plant species in the Provinces of Jiangxi, Guangdong, Hubei and Guizhou since the first record in 1985. However, the existence of unique morphological features in some species within the genus Phasmatidae led to the emergence of a new genus, which included new taxa and new nomenclature of *Medaurini* (Phasmatidae: Clitumninae) from China (Ho, 2021), *Cretophasmomima traceyae* sp. *nov*. (Phasmatodea: Susumanioidea) in southern England (Hennemann, 2020), *Diapheromera arena n.*sp. *t* (Phasmatodea: Diapheromeridae: *Diapheromera*) from western Texas and New Mexico (Stidham and Stidham, 2018), the genus *Parapachymorpha Brunner von Wattenwyl,* 1893 (Phasmatidae, Phasmatidae, Clitumninae) from Laos, etc. At present, the control methods and technologies used for Phasmatidae pests mainly include physical methods, chemical methods and biological methods. Although chemicals are an effective control method, they will lead to drug resistance and pesticide residues by repeated use. Therefore, biological control measures are more popular to control these pests. In this paper, the sequence analysis of the mitochondrial genome of *R. irregulatiter dentatus* also provides a reference for the use of biotechnology to control forest pests. On the other hand, despite Phasmatidae as a phytophagous and predatory species with important agricultural value, their evolutionary relationships remained ineffectively explored. The mitogenome data of *R. irregulatiter dentatus* would be useful for further genetic studies, phylogenetic analysis, taxonomic resolution and pest control of Phasmatidae insects.

## Data Availability

The datasets presented in this study can be found in online repositories. The names of the repository/repositories and accession number(s) can be found in the article/supplementary material.
